# Transcriptome Analysis of Testicular Aging in Mice

**DOI:** 10.3390/cells10112895

**Published:** 2021-10-26

**Authors:** Gwidong Han, Seong-Hyeon Hong, Seung-Jae Lee, Seung-Pyo Hong, Chunghee Cho

**Affiliations:** School of Life Sciences, Gwangju Institute of Science and Technology, Gwangju 61005, Korea; gdhan@gm.gist.ac.kr (G.H.); alchemist9737@gist.ac.kr (S.-H.H.); lsj9317@gist.ac.kr (S.-J.L.); hongsp8092@gm.gist.ac.kr (S.-P.H.)

**Keywords:** spermatogenesis, testis, aging, long non-coding RNA, testicular aging, andropause

## Abstract

Male reproductive aging, or andropause, is associated with gradual age-related changes in testicular properties, sperm production, and erectile function. The testis, which is the primary male reproductive organ, produces sperm and androgens. To understand the transcriptional changes underlying male reproductive aging, we performed transcriptome analysis of aging testes in mice. A total of 31,386 mRNAs and 9387 long non-coding RNAs (lncRNAs) were identified in the mouse testes of diverse age groups (3, 6, 12, and 18 months old) by total RNA sequencing. Of them, 1571 mRNAs and 715 lncRNAs exhibited changes in their levels during testicular aging. Most of these aging-related transcripts exhibited slight and continuous expression changes during aging, whereas some (9.6%) showed larger expression changes. The aging-related transcripts could be classified into diverse expression patterns, in which the transcripts changed mainly at 3–6 months or at 12–18 months. Our subsequent in silico analysis provided insight into the potential features of testicular aging-related mRNAs and lncRNAs. We identified testis-specific aging-related transcripts (121 mRNAs and 25 lncRNAs) by comparison with a known testis-specific transcript profile, and then predicted the potential reproduction-related functions of the mRNAs. By selecting transcripts that are altered only between 3 and 18 months, we identified 46 mRNAs and 34 lncRNAs that are stringently related to the terminal stage of male reproductive aging. Some of these mRNAs were related to hormonal regulation. Finally, our in silico analysis of the 34 aging-related lncRNAs revealed that they co-localized with 19 testis-expressed protein-coding genes, 13 of which are considered to show testis-specific or -predominant expression. These nearby genes could be potential targets of *cis*-regulation by the aging-related lncRNAs. Collectively, our results identify a number of testicular aging-related mRNAs and lncRNAs in mice and provide a basis for the future investigation of these transcripts in the context of aging-associated testicular dysfunction.

## 1. Introduction

Male reproductive aging, or andropause, refers to age-related changes in male reproductive integrity, including sperm production and erectile function [1]. Unlike menopause resulting from female reproductive aging, which is relatively abrupt, male reproductive aging is accompanied by slow changes in function [1,2]. These gradual dysfunctional changes occur mostly in the testis, which is the main male reproductive organ and is responsible for producing sperm and hormones [1,2,3]. Slow and progressive decreases in the testicular mass and testosterone level, erectile dysfunction, and tubular sclerosis are observed during human andropause [2,4].

Spermatogenesis is the unique, complex, and tightly regulated process of male germ cell development [5]. It includes successive mitotic division, meiosis, and post-meiotic phases through which spermatogonial stem cells (SSCs) are differentiated into spermatocytes and spermatids respectively. A highly organized intrinsic genetic network is important for spermatogenesis [5]. To elucidate the molecular nature of the intrinsic program of spermatogenesis, various investigations have sought to identify and characterize relevant protein-coding genes [6] and noncoding RNAs, such as microRNAs (miRNAs) [7], piwi-interacting RNAs (piRNAs) [8], and long non-coding RNAs (lncRNAs) [9,10,11,12,13,14,15]. LncRNAs could function as novel regulatory molecules which interact with DNA, RNA, and proteins. They are implicated in various biological process, including differentiation and development. Our group previously found that the testis harbors many tissue-specific lncRNAs [16]. We recently reported that the testicular germ cell-specific lncRNA, *Teshl*, functions to regulate the expression of sex chromosome genes and maintain the quality of Y chromosome-bearing sperm [17].

Testicular aging has been studied in humans [1,2,3,4,17,18,19], as well as in murine [20] and canine species [21]. In humans and canines, decreases in Leydig cell populations are commonly observed [19,21]. In the case of mouse testicular aging, plasma testosterone level was significantly decreased in aged groups (over age 450 days). It is unclear whether there are changes in semen parameters, such as sperm concentration, motility, and morphology, during aging [22]. An integrative study with genotype tissue expression (GTEx) data identified 22 and 7 aging-associated coding (mRNA) and non-coding (lncRNA) genes, respectively, in human testes [18]. However, although numerous aging-related studies have been performed on mouse tissues including testes and various tissues of other species, no previous study has undertaken aging-related transcriptomic profiling of whole mouse testes in a comprehensive manner.

Here, we identified and profiled mRNAs and lncRNAs associated with mouse testicular aging through total RNA sequencing analysis. To comprehensively investigate the expression changes of transcripts during all periods of aging, we analyzed the testicular transcripts obtained at postnatal months 3, 6, 12, and 18. We newly identified aging-related transcripts, observed that they typically exhibited slight and gradual expression changes, classified them into a number of different expression patterns, and assessed the potential features of the aging-related mRNAs and lncRNAs. To the best of our knowledge, this is the first study seeking to identify and characterize mRNAs and lncRNAs related to testicular aging in mice. Our study provides inclusive transcriptomic information that should facilitate future studies on the testicular dysfunction that occurs with age in mammalian species.

## 2. Materials and Methods

### 2.1. Animals and RNA Preparation

We used C57BL/6 male mice representing four age groups: postnatal 3, 6, 12, and 18 months old. Three life phases (3–6 months of age for ‘mature adult’, 10–15 months of age for ‘middle-aged’, and 18–24 months of age for ‘old’) have been suggested for aging studies in mice (https://www.jax.org/research-and-faculty/research-labs/the-harrison-lab/gerontology/life-span-as-a-biomarker, accessed on 3 September 2019). All mice were provided by the Korea Basic Science Institute (KBSI, Gwangju, Korea). It is unclear whether mice of each age group were from the same litter. Each experiment was performed in triplicate. Testes were obtained from mice and immediately cryopreserved with liquid nitrogen and stored at −80 °C. Total RNA was isolated. Each experiment involving animals was conducted in accordance with the Korean Food and Drug Administration (KFDA) test guidelines. The protocols were reviewed and approved by the Institutional Animal Care and Use Committees (IACUC) of Gwangju Institute of Science and Technology (GIST) (permit number: GIST-2019-097).

### 2.2. Total RNA Sequencing

Sequencing libraries were prepared using a TruSeq RNA Sample Prep kit v2 (Illumina, San Diego, CA, USA) according to the provided protocol. Library preparation involved removing ribosomal RNA with a Ribo-Zero rRNA Removal Kit (Illumina), and then performing random fragmentation and cDNA synthesis. Each sample library was subjected to paired-end high-throughput RNA sequencing using a HiSeq 4000 system (Illumina). The raw reads obtained from high-throughput sequencing were subjected to quality control and preprocessing. Alignment was performed with HISAT2 against the UCSC mm10 reference genome. Expression profiles were obtained with the FPKM (fragments per kilobase of exon per million fragments) method. To determine the expression changes of transcripts, we used fold-change with pseudocount value (0.001). The sequencing data were uploaded to the Gene Expression Omnibus (GEO) database at the National Center for Biotechnology Information (NCBI) under GEO accession number GSE175633.

### 2.3. Tissue Expression Estimation

We previously performed microarray analysis of five different mouse tissues (brain, heart, kidney, liver, and testis) to identify tissue-specific expression of mRNAs and lncRNAs [16]. Here, we identified age-related mRNAs and lncRNAs showing apparent testis-specific expression by comparing the previous tissue expression data with the newly identified age-related transcripts. We converted the transcript IDs of the age-related and testis-specific transcripts into ENSEMBL gene IDs using the DAVID gene ID conversion tool.

### 2.4. Investigating the Potential Features of Aging-Related Transcripts

We selected aging-related mRNAs and lncRNAs to investigate their potential features. A protein-protein network was constructed with the 46 mRNAs using the Search Tool for the Retrieval of Interacting Genes/Proteins (STRING) [23]. To investigate *cis*-regulatory targets of the 34 aging-related lncRNAs, we used Genomic Regions Enrichment of Annotations Tool (GREAT) to identify their neighboring genes (Basal plus extension; proximal, 5 kb upstream and 1 kb downstream; distal, up to 1000 kb) [24].

## 3. Results

### 3.1. Identification of mRNAs and lncRNAs in Mouse Testes during Aging

To comprehensively identify transcripts expressed in mouse testes at different ages, we prepared testes from C57BL/6 mice representing four age groups: postnatal 3 months old (3M), 6 months old (6M), 12 months old (12M), and 18 months old (18M). We performed total RNA sequencing analysis of testicular tissues using an Illumina HiSeq 4000. The raw data comprised 65 to 90 million total reads, over 95% of which were clean reads (Table 1). These data were deposited to the Gene Expression Omnibus (GEO) under GEO accession number GSE175633. The testicular transcripts obtained from the RNA sequencing data were processed into individual transcripts using StringTie [25]. A total of 71,967 transcripts were assembled from the entire set of raw RNA sequences.

**Figure 1:**
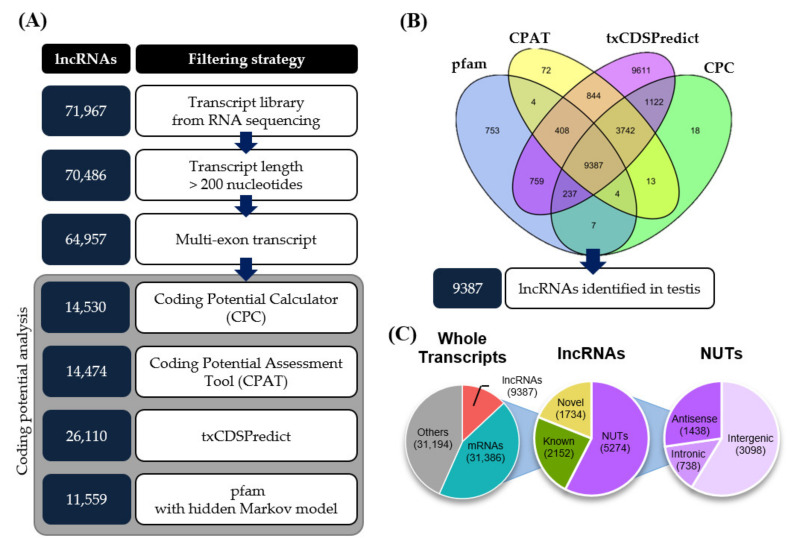
Pipeline for identifying testis-expressed lncRNAs from total RNA sequencing data by in silico analysis. (**A**) Filtering strategy for identifying lncRNAs from total RNA sequencing data. (**B**) Intersection of coding potential analysis tools (CPC, CPAT, txCDSPredict, and pfam with hidden Markov model). (**C**) Genome-wide composition of transcripts identified in aged mouse testis using the following class codes from CuffCompare. “Others” represents ncRNAs with single-exon sequences and sequences shorter than 200 nt.

From the total set of individual transcripts, 31,386 transcripts were identified as mRNAs based on our analysis using the MGI (Mouse Genome Informatics) database and GffCompare [26]. Total RNA sequencing can also provide information on the expression profiles of non-coding RNAs. To identify lncRNAs from the transcript assembly, we designed an in silico pipeline (Figure 1A). First, based on the definition of an lncRNA, we selected transcripts longer than 200 nucleotides (nt). We then filtered out single-exon transcripts, which yielded 64,957 multi-exon transcripts, to eliminate experimental artifacts and background noise. As a consequence, single-exon lncRNAs were excluded from our lists. Finally, we assessed the coding potential of these transcripts using CPC (Coding Potential Calculator) [27], CPAT (Coding Potential Assessment Tool) [28], txCDSPredict (provided by kentUtils), and HMMSearch (provided by HMMER) against the pfam database [29] (Figure 1B). Of the transcripts, 9387 were commonly evaluated as non-coding sequences by these tools and were thus considered to be testicular lncRNAs. Further classification of these lncRNAs revealed that 2152 were known non-coding transcripts, 1734 were novel isoforms of known transcripts, and the remaining 5274 were novel unannotated transcripts (NUTs) (Figure 1C).

### 3.2. Global Expression and Transcriptomic Features of mRNAs and lncRNAs Expressed in Mouse Testes during Aging

**Figure 2:**
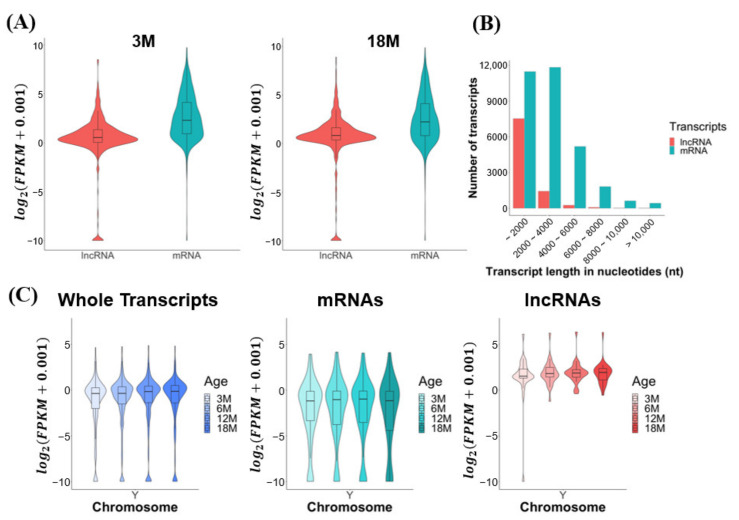
Transcriptomic features of mouse testes during aging. The data were obtained from total RNA sequencing. (**A**) Global expression level distribution of lncRNAs and mRNAs in the 3M and 18M mouse age groups. (**B**) Length distributions of lncRNAs and mRNAs. (**C**) Global expression level distributions of whole transcripts, mRNAs, and lncRNAs for the Y chromosome. Expression levels are presented as log2(FPKM + 0.001).

We characterized the expression and transcriptomic features of the mRNA and lncRNA transcripts identified by our total RNA sequencing. The average expression levels of mRNAs were modestly higher than those of lncRNAs (average 1.32-fold) in both young (3M) and old (18M) age groups (Figure 2A). Most of the lncRNAs (69%) varied in length from 200 to 2000 nt, and the majority of the mRNAs (74.2%) ranged from 200 to 4000 nt (Figure 2B). The expression levels of total transcripts, mRNAs, and lncRNAs were found to be similar among the age groups, from 3M to 18M, for all mouse chromosomes except for the Y chromosome (Supplementary Figure S1): the average expression levels of total transcripts and lncRNAs, but not mRNAs, increased slightly from 3M to 18M for the Y chromosome (Figure 2C).

### 3.3. Aging-Related Expression Patterns of mRNAs and lncRNAs

**Figure 3:**
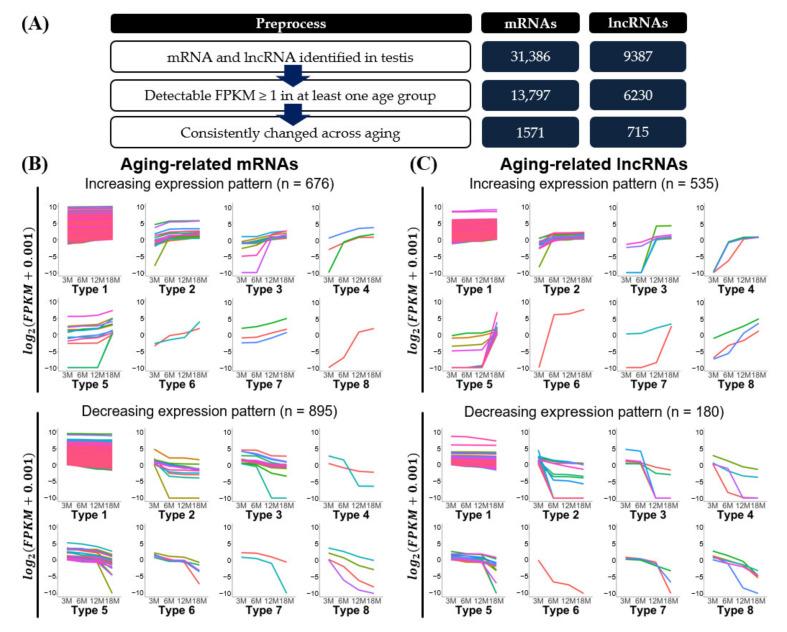
Expression change patterns of aging-related transcripts. (**A**) Preprocessing of identified mRNAs and lncRNAs was used to classify expression patterns. Expression change patterns of aging-related (**B**) mRNAs and (**C**) lncRNAs are described in line plots. Discrete colored lines represent each transcript. Expression levels are presented as log_2_(FPKM + 0.001). Expression patterns were classified into eight different types. Details of the expression pattern classification are shown in Table 2 and Supplementary Tables S2–S5.

Although the overall expression levels of transcripts were similar among the age groups, we expected that sets of transcripts would show changes during aging. To characterize aging-related mRNAs and lncRNAs in depth, we analyzed their expression patterns during testicular aging. For data preprocessing of the identified mRNAs and lncRNAs, we set the expression level criteria to select transcripts with FPKM ≥ 1 in at least one age group. This analysis yielded 13,797 mRNAs and 6230 lncRNAs. In addition, biological aging is accompanied with long and gradual accumulation of genetic damage [30]. To detect the result of accumulated damage and/or physiological changes, we investigate the continuous expression changes in testicular aging. We found that 1571 mRNAs and 715 lncRNAs showed a continuous increase or decrease, respectively, from 3M to 18M. Evaluation of the expression changes was based on the average values of three testicular samples in each age and statistical significance was not considered in this analysis (Figure 3A and Supplementary Table S1).

To further analyze the expression patterns of transcripts exhibiting continuous gradual increases or decreases during testicular aging (3M, 6M, 12M, and 18M), we classified the mRNAs and lncRNAs by their degree of expression change across three consecutive age-comparison groups: 3M to 6M, 6M to 12M, and 12M to 18M. We considered transcripts with log_2_(|Fold change|) ≥ 1.2 to exhibit a “substantial expression change”, as opposed to transcripts exhibiting a “slight change” over that period. From this analysis, we classified the mRNAs and lncRNAs into each eight groups representing increasing or decreasing expression patterns (Figure 3B,C and Table 2).

The classified expression patterns revealed several features. The majority (over 80%) of the transcripts [93.1% of mRNAs (1463 of 1571) and 85.8% of lncRNAs (614 of 715)] were classified as type 1, the members of which commonly showed very slight changes in expression level between the age groups (Figure 3B,C and Table 2). The remaining transcripts (108 mRNAs and 111 lncRNAs) distributed to types 2–8, each of which included mRNAs and lncRNAs with increasing or decreasing patterns. The types can be divided into two groups, one comprising types 2, 3, and 5 and one comprising types 4, 7, and 8. The members of types 2, 3, and 5 showed a substantial expression change in a single age-comparison group (3M–6M, 6M–12M, or 12M–18M) (Figure 3B,C and Table 2). For example, members of type 2 exhibited a substantial expression change only between 3M and 6M (28 mRNAs and 41 lncRNAs) (Table 2). In contrast, members of types 4, 7, and 8 exhibited substantial expression changes in more than one age-comparison group (Figure 3B,C and Table 2). For instance, members of type 4 showed substantial expression changes at both 3M–6M and 6M–12M groups (5 mRNAs and 7 lncRNAs) (Table 2). Our analysis revealed that more transcripts showed a substantial expression change in only a single age-comparison group (174 transcripts) than in multiple age-comparison groups (36 transcripts). Interestingly, we observed that major expression changes during aging tend to be limited to the 6 month period (maximum 12M to 18M). We obtained information on gene ontology (GO) enriched in each classified expression pattern (Supplementary Tables S6 and S7). Spermatogenesis (GO:0007283) and single fertilization (GO:0007338) were found to be enriched in increasing expression patterns with substantial expression changes (Types 2–8) (Supplementary Table S6). GO terms with extracellular exosome (GO:0070062) and hydrolase activity (GO:0016787) were enriched in decreasing expression patterns having substantial expression changes (Types 2–8) (Supplementary Table S7).

### 3.4. Testis-Specific Expression of Aging-Related mRNAs and lncRNAs

To investigate the potential effect of aging on the transcriptome related to testis-specific features, such as spermatogenesis, we identified aging-related mRNAs and lncRNAs that are thought to be exclusively expressed in testes. In a previous study, we identified testis-specific mRNAs and lncRNAs from microarray analysis [16]. Using Ensembl Gene IDs, we herein compared aging-related transcripts found in the present study (1571 mRNAs and 715 lncRNAs of types 1–8) with the previously identified testis-specific transcripts. We identified at least 121 mRNAs and 25 lncRNAs as putative testis-specific aging-related transcripts (Supplementary Table S8). For the mRNAs, we used DAVID to perform function-enrichment analysis and predict their functional roles. We obtained information on gene ontology (GO) terms with *p*-value ≤ 0.05, and found that the 121 mRNAs were related to male reproductive components and processes, such as acrosomal vesicle (GO:0001669), male meiosis (GO:0007140), spermatogenesis (GO:0007283), and sperm-egg recognition (GO:0035036) (Supplementary Table S9). To provide more precise information on mRNAs with testis specificity, we cross-checked testis-specific aging-related mRNAs with mouse ENCODE RNA sequencing data (http://genome.ucsc.edu/ENCODE, accessed on 13 October 2021) containing global tissue expression data. With the TissueEnrich tool [31], we narrowed down 121 putative testis-specific mRNAs to 77 mRNAs showing further testis-enriched expression (Supplementary Figure S3). Thus, 4.9% (77 of 1571) of the aging-related mRNAs were predicted to be expressed in a testis-specific manner, suggesting that their expression changes could affect male reproductive functions during aging. 

### 3.5. Potential Features of Aging-Related Transcripts Showing Changes between 3M and 18M

**Figure 4:**
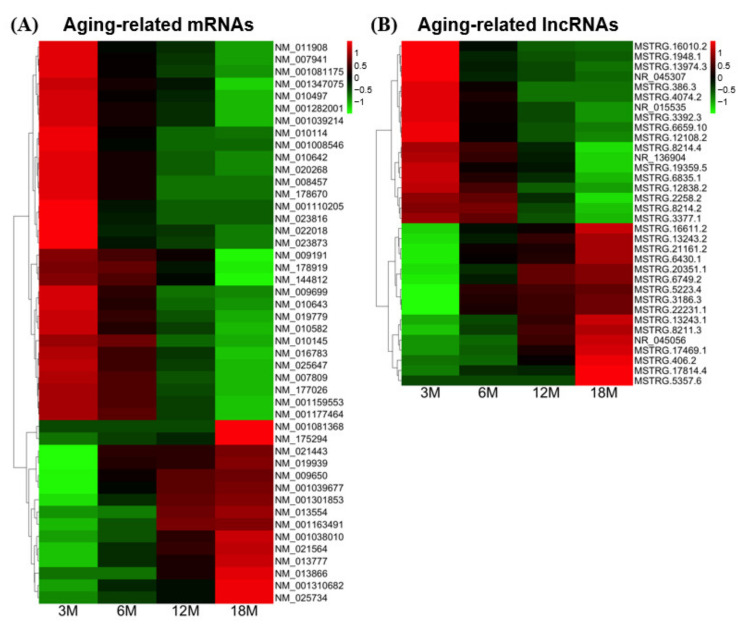
Heatmap showing the relative expression levels of the 46 significant aging-related mRNAs (**A**) and 34 significant aging-related lncRNAs (**B**) that exhibited differential expression in the four age groups. Red and green colors indicate higher and lower expression levels, respectively. Transcript IDs are presented on the right.

To further investigate the potential functional features of aging-associated transcripts in testes, we selected transcripts that were more stringently related to the terminal stage of aging. Unlike our analysis of transcripts whose expression levels showed continuous increases or decreases across all age groups (Figure 3B,C and Table 2), we focused on transcripts that showed changes only between 3M (youngest age) and 18M (oldest age). These transcripts were chosen using DESeq2 with the criteria of a significant expression change (*p*-value ≤ 0.05 for three biological replicates) and a particular fold-change (log_2_(|Fold change|) ≥ 1) between the 3M and 18M age groups. This strategy selected 46 mRNAs and 34 lncRNAs (Figure 4), 55% of which (44/80) were members of types 2–8 and showed substantial expression changes (Figure 3B,C and Table 2). The remaining transcripts (36/80) did not show substantial expression changes between the studied age-group pairs (type 1), but showed significant changes between 3M and 18M.

We investigated the potential functional features of these transcripts (Table 3). For the mRNAs, 71% (33/46) were members of types 2–8 and showed substantial expression changes; in contrast, only 32.4% (11/34) of the lncRNAs were members of these types. We constructed a protein–protein interaction (PPI) network of the mRNAs predicted to be strongly associated with testicular aging [23]. Through a STRING-based PPI network analysis, we identified potential hub genes that were predicted to have more than one connection with other aging-related genes (Supplementary Figure S4). Notably, some of these hub genes are known to be involved in male reproduction. For example, progesterone receptor membrane component 1 (*Pgrmc1*) is implicated in the proliferation of male germ cells, as found in a conditional deletion study [32]. Cytochrome P450, family 7, subfamily a, polypeptide 1 (*Cyp17a1*) and cytochrome P450, family 11, subfamily a, polypeptide 1 (*Cyp11a1*) are cytochrome P450 family genes [33]. They encode proteins that are located in the inner mitochondrial membrane and endoplasmic reticulum, respectively, and participate in steroidogenesis. Finally, the expression level of kallikrein 1-related peptidase b27 (*Klk1b27*) is known to be regulated by testosterone in testis [34].

### 3.6. Potential Cis-Regulatory Targets of Aging-Related lncRNAs

Many functional lncRNAs are known to act in a *cis*-regulatory manner. To investigate the potential *cis*-regulatory targets of the 34 aging-related lncRNAs described above (Figure 4), we analyzed nearby genomic regions using Genomic Regions Enrichment of Annotations Tool (GREAT) [24]. This strategy identified 56 protein-coding genes that were located in genomic regions near the identified aging-related lncRNAs, and could thus be potential *cis*-regulatory targets. Indeed, five of these neighboring genes were found to show changes during aging: The mRNA expression levels of four decreased (*Agtpbp1*, *Suds3*, *2410089E03Rik*, and *Tex14*) with age, whereas one increased (*4921509C19Rik*) (Figure 4). LncRNAs for three mRNAs (*Suds3*, *2410089E03Rik* and *Tex14*) also showed decreases in level during aging. In contrast, the expression pattern (increase or decrease in level) of lncRNAs for the other two mRNAs (*Agtpbp1* and *4921509C19Rik*) was opposite to that of the mRNAs. We also examined the testis expression of the neighboring genes using the ENCODE database, and found that 21 of them are known to be expressed in testes (Table 4). Of these testicular genes, 13 exhibit testis-predominant (6 genes) or -specific (7 genes) expression. Finally, we performed functional enrichment tests with the potential testis-specific and -predominant *cis*-regulatory target genes. We found that three of the genes were related to the GO terms of cellular response to hormone stimulus (GO:0032870: *4921509C19Rik*, and *Gm14717*) and protein phosphorylation (GO:0006468: *4921509C19Rik*, *Gm14717*, and *Tex14*).

## 4. Discussion

In the present study, we comprehensively investigated transcriptional changes associated with biological aging in adult mouse testis. In previous reports, sperm parameters and the morphology of testicular somatic cells were analyzed in consecutive age groups of various mammalian species, including humans [1,2,3,4,19], canines [21], and murine species [17]. Further studies investigated aging-related testicular changes in the context of epigenetic modification and gene expression [18,20]. Here, we used total RNA sequencing to reveal the profiles of transcripts, including both mRNAs and lncRNAs, in mouse testes of multiple age groups (3M, 6M, 12M, and 18M). We identified a total of 31,386 mRNAs and 9387 lncRNAs in the testes. Notably, the number of testicular lncRNAs identified by our total RNA sequencing analysis is similar to those found previously using microarray analysis (8265~14,256 lncRNAs) and single-cell RNA sequencing analysis (9431 lncRNAs) in mouse testes [16,35,36,37]. Nonetheless, library preparation with TruSeq RNA Library Prep Kit v2, which is non-stranded, limited our analysis in identifying antisense RNAs exhaustibly. Further study using stranded RNA library may extend our profiling of antisense lncRNAs. Importantly, we newly identified 1571 mRNAs and 715 lncRNAs associated with testicular aging in mice (Figure 1 and Figure 2).

Our genome-level analysis revealed that the total transcripts and lncRNAs expressed from the Y chromosome increased slightly during testicular aging (Figure 2). It was previously reported that histone modifications were altered on the peri-chromocenter, predicted to be putative sex chromosomes, of aged human spermatogenic cells [20]. It is possible that the observed increase in lncRNAs reflects transcriptional noise derived from cellular senescence [30,38]. Further studies are warranted to examine aging-related transcriptional changes at the chromosome level.

We investigated the expression patterns of the aging-associated transcripts (1571 mRNAs and 715 lncRNAs) in depth, seeking to elucidate the age(s) at which major transcriptional changes occur in the testis. The degree of differential gene expression during aging in mice has been found to vary by tissue type [39]. Here, we observed that the majority of aging-associated genes in testes showed only slight changes between the age groups. It should be noted that these changes were not statistically significant. Thus, the expression changes could be artefacts due to differences among animals and/or deviation between RNA sequencing analyses. Alternatively, this may represent the nature of aging that shows gradual and slight expression changes hard to detect experimentally. To confirm the gene expression changes observed in this study requires further investigation. In this regard, gene expression analysis of specific testicular cell types, instead of whole testes, is necessary, since cell-type specific expression differences may be hidden. Previously, gene expression analysis of spermatocytes in rats during aging revealed alteration of genes related to cell adhesion [40].

The analyses showing larger (herein termed “substantial”) changes tended to be found in the 3M–6M and 12M–18M groups (Table 2). This is in line with previous reports that the largest numbers of expression-altered genes were observed during periods considered middle-to-old age (12M–18M) in gonadal adipose tissue (GAT) and subcutaneous adipose tissue (SCAT) in mice [30]. Perhaps gene expression alterations that emerge from middle age could affect testicular function in late life. Transcriptional analysis of an age group older than 18M might be necessary to explore this possibility.

Testis-specific genes play important roles in male reproduction [5,6,16]. Interestingly, the testis contains the largest number of tissue-specific mRNAs and lncRNAs. We recently reported that the testis-specific lncRNA, *Teshl*, promotes the expression of genes on the Y chromosome and thereby regulates the offspring sex ratio in mice [17]. In the present study, we identified 121 mRNAs and 25 lncRNAs as being testis-specific aging-related transcripts. Function-enrichment analysis of these mRNAs revealed that some are related to male reproductive functions. Regarding the potential *cis*-regulatory targets of aging-related lncRNAs, one of the candidates is *Tex14*. This gene is required for intercellular bridge formation in spermatogenic cells and essential for male mouse fertility [41]. The observed decrease in level of *Tex14* in our analysis suggests a possible link of the gene to abnormal spermatogenesis during testicular aging. Another interesting novel candidate is 4921509C19Rik, which showed an increase in expression level during testicular aging. This gene has been predicted to play a role in sperm motility (https://www.uniprot.org/uniprot/Q8C0X8, accessed on 13 October 2021). It should be noted that search of potential *cis*-regulatory targets was performed with mild criteria (1 Mb distance), requiring further investigation for *cis*-regulatory relationship. These testis-specific aging-related transcripts could be causative genes for aging and/or reflect male reproductive dysfunction due to testicular aging.

The decline of male reproductive hormone levels is a main cause of andropause, and aging in mammals is associated with a decrease in the testosterone level [2,4,19]. Here, we observed aging-related expression changes of genes related to steroid hormone synthesis and reactions, such as *Cyp17a1*, *Cyp11a1*, and *Klk1b27* [33,34]. In the context of male reproductive aging, altered hormonal regulation is likely to affect the intrinsic regulation of gene expression in male germ cells.

Notably, most of the gene expression changes observed herein during testicular aging were slight and gradual. The majority of identified transcripts were members of type 1, showing small changes in expression level between the age groups. This is consistent with the gradual, physiological dysfunction seen in andropause, which contrasts with the more abrupt alterations seen during female menopause. The nature of aging appears to involve transcriptional changes of small proportions of genes, as shown in human kidney [42], human brain [43,44], monkey skeletal muscle [45] (~4%), fruit fly heart (~3%), human Achilles tendon [46], human retina [47,48], rodent brain [49], rodent skeletal muscle [50], rodent liver [51], and rodent heart (≤2%) [39,50,52,53]. In the present study, the aging-related testicular transcripts accounted for 5.6% (2292) of the total identified transcripts (40,773). These observations suggest that the transcriptional landscape of aging should be elucidated in a sophisticated and precise way. In this regard, it could be important to investigate a possibility that some gene expression changes observed during aging are attributable to changes in cellular composition. In fact, a recent study showed that aging could affect differentiation of mouse SSCs, resulting in transcriptomic alterations [54].

In conclusion, we herein analyzed the transcriptional changes of mRNAs and lncRNAs during mouse testicular aging, and provide a comprehensive profile of the aging-associated transcripts. Most of the identified transcripts showed modest and gradual transcriptional changes. We classified the aging-related testicular transcripts into eight types based on their expression patterns. Further analyses provided additional insights, including potential features of aging-related mRNAs and potential *cis*-regulatory targets of aging-related lncRNAs. The present findings improve our understanding of the molecular mechanisms underlying testicular dysfunction in aging and should facilitate future investigations into the transcriptional signature of male reproductive aging.

## Figures and Tables

**Table 1:** Raw data summary for total RNA sequencing of mouse testis from different age groups.

Samples	Total Reads	Total Read Bases ^1^	Q20(%) ^2^	Q30(%) ^3^
3M-1	67,091,602	6,776,251,802	98.6	96.17
3M-2	73,106,236	7,383,729,836	98.5	95.93
3M-3	73,869,148	7,460,783,948	98.63	96.24
6M-1	66,020,784	6,668,099,184	98.48	95.75
6M-2	65,090,562	6,574,146,762	98.56	96.09
6M-3	71,624,278	7,234,052,078	98.59	96.15
12M-1	75,862,548	7,662,117,348	98.67	96.27
12M-2	69,647,216	7,034,368,816	98.62	96.16
12M-3	79,299,106	8,009,209,706	98.62	96.18
18M-1	72,565,298	7,329,095,098	98.47	95.89
18M-2	67,057,420	6,772,799,420	98.67	96.27
18M-3	90,942,020	9,185,144,020	98.35	95.33

^1^ Total read bases: the number of bases sequenced in RNA sequencing, which was derived by the total reads × read length. ^2^ Q20: the ratio of bases having a Phred Quality score over 20. ^3^ Q30: the ratio of bases having a Phred Quality score over 30.

**Table 2:** Summary of aging-related transcript expression pattern in mouse testes.

	Type	Substantial Expression Change ^1^			mRNAs ^2^	lncRNAs ^2^	Total ^2^
		3M to 6M	6M to 12M	12M to 18M			
Increased	1				629	481	1110
	2	+			17	17	34
	3		+		9	7	16
	4	+	+		3	3	6
	5			+	12	21	33
	6	+		+	2	1	3
	7		+	+	3	2	5
	8	+	+	+	1	3	4
Decreased	1				834	123	957
	2	+			11	24	35
	3		+		13	5	18
	4	+	+		2	4	6
	5			+	24	14	38
	6	+		+	5	1	6
	7		+	+	2	3	5
	8	+	+	+	4	6	10
Total					1571	715	2286

^1^ The “+” mark represents a substantial expression change between age groups. ^2^ The numbers of aging-related mRNAs, lncRNAs, and total transcripts are indicated.

**Table 3:** Expression changes and classified patterns of aging-related mRNAs.

Transcript ID	Gene Symbol	Average FPKM				3M–18M*p*-Value ^1^	Expression Pattern	
		3M	6M	12M	18M		Inc/Dec ^2^	Type
NM_001110205	*Acvr1*	1.16	0.26	0.01	0.00	8.98 × 10^−58^	Decreased	8
NM_010114	*Klk1b22*	4.36	1.63	0.33	0.14	4.52 × 10^−4^	Decreased	8
NM_013866	*Zfp385a*	0.00	0.01	1.59	3.40	4.12 × 10^−6^	Increased	8
NM_020268	*Klk1b27*	12.33	5.92	1.98	0.93	3.96 × 10^−56^	Decreased	8
NM_177026	*Tmcc3*	1.81	1.39	0.51	0.00	4.62 × 10^−7^	Decreased	7
NM_001081175	*Itpkb*	2.85	1.23	0.62	0.01	1.70 × 10^−77^	Decreased	6
NM_022018	*Fam129a*	3.36	0.92	0.75	0.09	7.93 × 10^−6^	Decreased	6
NM_025734	*Kcng4*	0.09	0.77	1.28	3.50	6.89 × 10^−5^	Increased	6
NM_001039214	*Mex3c*	12.72	7.02	4.52	0.46	1.13 × 10^−7^	Decreased	5
NM_001081368	*Tbccd1*	0.15	0.15	0.16	1.01	3.88 × 10^−7^	Increased	5
NM_001282001	*Rbl2*	1.92	1.02	0.62	0.00	9.08 × 10^−5^	Decreased	5
NM_001347075	*Xpnpep3*	2.60	1.48	1.13	0.05	8.90 × 10^−72^	Decreased	5
NM_009191	*Clpb*	10.02	8.83	7.53	2.69	3.70 × 10^−4^	Decreased	5
NM_010497	*Idh1*	5.08	2.89	2.34	0.92	4.03 × 10^−7^	Decreased	5
NM_016783	*Pgrmc1*	36.82	28.19	16.46	6.24	1.74 × 10^−20^	Decreased	5
NM_025647	*Cmpk1*	5.82	4.32	2.46	0.99	6.39 × 10^−117^	Decreased	5
NM_144812	*Tnrc6b*	1.15	1.02	0.70	0.04	2.51 × 10^−10^	Decreased	5
NM_175294	*Nucks1*	0.24	0.40	0.50	1.37	3.43 × 10^−5^	Increased	5
NM_178919	*Lmf2*	8.59	8.09	5.64	2.06	1.52 × 10^−3^	Decreased	5
NM_001163491	*Sema4a*	0.12	0.49	1.49	1.55	5.96 × 10^−17^	Increased	4
NM_008457	*Klk1b8*	6.27	2.70	0.01	0.01	9.23 × 10^−11^	Decreased	4
NM_001038010	*Kat2a*	0.62	1.18	2.49	3.72	1.12 × 10^−3^	Increased	3
NM_009699	*Aqp2*	2.79	1.82	0.76	0.62	3.70 × 10^−10^	Decreased	3
NM_010642	*Klk1b21*	16.36	8.22	3.01	1.72	4.60 × 10^−6^	Decreased	3
NM_010643	*Klk1b24*	16.44	9.93	3.53	1.92	6.06 × 10^−48^	Decreased	3
NM_013554	*Hoxd10*	0.50	0.57	1.45	1.59	5.51 × 10^−5^	Increased	3
NM_001039677	*Slc30a2*	1.37	4.14	5.35	5.77	2.19 × 10^−87^	Increased	2
NM_001301853	*Stk11*	0.24	1.54	2.87	3.15	1.90 × 10^−20^	Increased	2
NM_019939	*Mpp6*	11.41	37.46	39.21	46.83	4.51 × 10^−11^	Increased	2
NM_021443	*Ccl8*	0.72	2.28	2.32	2.70	4.32 × 10^−4^	Increased	2
NM_021564	*Fetub*	0.40	1.59	2.61	3.71	1.63 × 10^−12^	Increased	2
NM_023816	*Ankrd36*	2.21	0.82	0.47	0.45	1.31 × 10^−4^	Decreased	2
NM_023873	*Cep70*	1.59	0.75	0.58	0.41	1.87 × 10^−4^	Decreased	2
NM_001008546	*Tardbp*	1.29	0.69	0.44	0.42	2.37 × 10^−5^	Decreased	1
NM_001159553	*H13*	1.62	1.37	0.90	0.53	5.98 × 10^−12^	Decreased	1
NM_001177464	*Zfp516*	1.43	1.26	0.86	0.57	7.70 × 10^−7^	Decreased	1
NM_001310682	*Prkcd*	0.66	1.20	1.36	2.52	1.22 × 10^−3^	Increased	1
NM_007809	*Cyp17a1*	77.11	65.52	40.81	28.29	1.12 × 10^−15^	Decreased	1
NM_007941	*Stx2*	8.06	5.07	4.08	2.82	2.63 × 10^−4^	Decreased	1
NM_009650	*Akap3*	148.84	274.92	318.80	330.13	1.21 × 10^−8^	Increased	1
NM_010145	*Ephx1*	32.38	30.42	17.68	14.66	2.59 × 10^−7^	Decreased	1
NM_010582	*Itih2*	3.83	2.76	1.97	1.27	8.63 × 10^−10^	Decreased	1
NM_011908	*Ubl3*	39.60	26.48	23.20	16.98	4.78 × 10^−6^	Decreased	1
NM_013777	*Akr1c12*	0.74	1.29	1.67	2.36	7.77 × 10^−21^	Increased	1
NM_019779	*Cyp11a1*	34.14	25.92	16.40	11.67	2.39 × 10^−7^	Decreased	1
NM_178670	*8030462N17Rik*	1.49	0.94	0.52	0.51	2.31 × 10^−4^	Decreased	1

^1^ 3M–18M *p*-value: *p*-value calculated by DESeq2 for the 3M vs. 18M age groups. ^2^ Inc/Dec: increased or decreased expression pattern.

**Table 4:** Testis-expressed potential *cis*-regulatory targets of aging-related lncRNAs identified by GREAT analysis.

Transcript ID	Transcript Locus	Nearby Gene	Nearby Gene Expression in Testis ^1^
MSTRG.2258.2	chr10:119413444–119453556	*Grip1*	testis predominant
MSTRG.6835.1	chr15:8350953–8351246	*Nipbl*	testis, brain
		*2410089E03Rik*	testis, CNS
MSTRG.3377.1	chr11:86811784–86816370	*Dhx40*	testis, bladder, CNS
MSTRG.12838.2	chr3:55055180–55084491	*Ccna1*	testis specific
MSTRG.1948.1	chr10:81383909–81395392	*Smim24*	testis, intestine
MSTRG.8214.2	chr16:59636945–59672993	*Arl6*	testis, CNS
MSTRG.8214.4	chr16:59636956–59672993		
NR_045307	chr19:45726555–45730558	*Npm3*	testis predominant
MSTRG.12108.2	chr2:151088381–151472250	*Gm14147*	testis specific
		*4921509C19Rik*	testis specific
MSTRG.406.2	chr1:73015899–73025507	*Tnp1*	testis specific
MSTRG.13243.2	chr3:100417896–100420833	*Fam46c*	testis specific
MSTRG.13243.1	chr3:100417891–100421080		
MSTRG.17469.1	chr6:125803352–125812378	*Ano2*	testis specific
MSTRG.21161.2	chr9:77357336–77363418	*Lrrc1*	testis predominant
MSTRG.22231.1	chrX:123448948–123456209	*Cldn34c2*	testis predominant
MSTRG.5223.4	chr13:49973191–49977078	*Gm906*	testis, kidney
MSTRG.3186.3	chr11:75588273–75594790	*Inpp5k*	testis, lung
MSTRG.6430.1	chr14:61648326–61668174	*Kcnrg*	testis predominant
MSTRG.16010.2	chr5:116985353–117004737	*Suds3*	testis, colon, ovary
MSTRG.3392.3	chr11:87405065–87555823	*Tex14*	testis specific
MSTRG.5357.6	chr13:59493404–59557347	*Agtpbp1*	testis predominant

^1^ Expression in mouse tissues was confirmed by reference to the mouse ENCODE project.

## Data Availability

The data presented in this study are openly available in Gene Expression Omnibus (GEO) database, at the National Center for Biotechnology Information (NCBI) under GEO accession number GSE175633.

## Supplementary Materials

The following are available online at https://www.mdpi.com/article/10.3390/cells10112895/s1: Figure S1. Global expression level distribution of whole transcripts, mRNAs and lncRNA in whole chromosomes. Expression levels are presented as log2(FPKM + 0.001); Figure S2. PCA plot between samples by DESeq2. (A) Aging-related mRNAs. (B) Aging-related lncRNAs; Figure S3. Tissue expression of 77 testis-enriched genes identified among testis-specific aging-related mRNAs by TissueEnrich, with mouse ENCODE expression data (BioProject, PRJNA66167); Figure S4. Protein-protein (PPI) network constructed by STRING database. Width of edge indicates the number of interactions detected in STRING and the color of each node presents each protein; Table S1. Aging-related mRNAs and lncRNAs showed a continuous increase or decrease from 3M to 18M. Class codes were identified with Cuffcompare (http://cole-trapnell-lab.github.io/cufflinks/cuffcompare/, accessed on 1 June 2020): known (class code ‘=’), novel (class code ‘j’), intergenic (class code ‘u’), intronic (class code ‘i’), and antisense (class code ‘x’); Table S2. Increasing mRNAs in classified expression patterns; Table S3. Decreasing mRNAs in classified expression pattern; Table S4. Increasing lncRNAs in classified expression patterns; Table S5. Decreasing lncRNAs in classified expression patterns; Table S6. Functional enrichment of mRNAs classified as increasing expression patterns. Gene ontology (GO) IDs and terms with *p*-value ≤ 0.05 and annotated ratio ≥ 5% are described. Category indicates GO aspects: molecular function (MF), cellular compartment (CC), and biological process (BP). GO enrichment data that do not satisfy criteria were omitted; Table S7. Functional enrichment of mRNAs as classified decreasing expression patterns. GO IDs and terms with *p*-value ≤ 0.05 and annotated ratio ≥ 5% are shown. Category indicates GO aspects; molecular function (MF), cellular compartment (CC), biological process (BP). GO enrichment data that do not satisfy criteria were omitted; Table S8. Putative testis-specific aging-related mRNAs and lncRNAs; Table S9. Functional enrichment of putative testis-specific aging-related mRNAs.
