# Supplementary figures and images for: Transcriptome Analysis of Testicular Aging in Mice
Source: Cells. 2021 Oct 26;10(11):2895. doi: 10.3390/cells10112895 (PMC8616291; doi:10.3390/cells10112895)

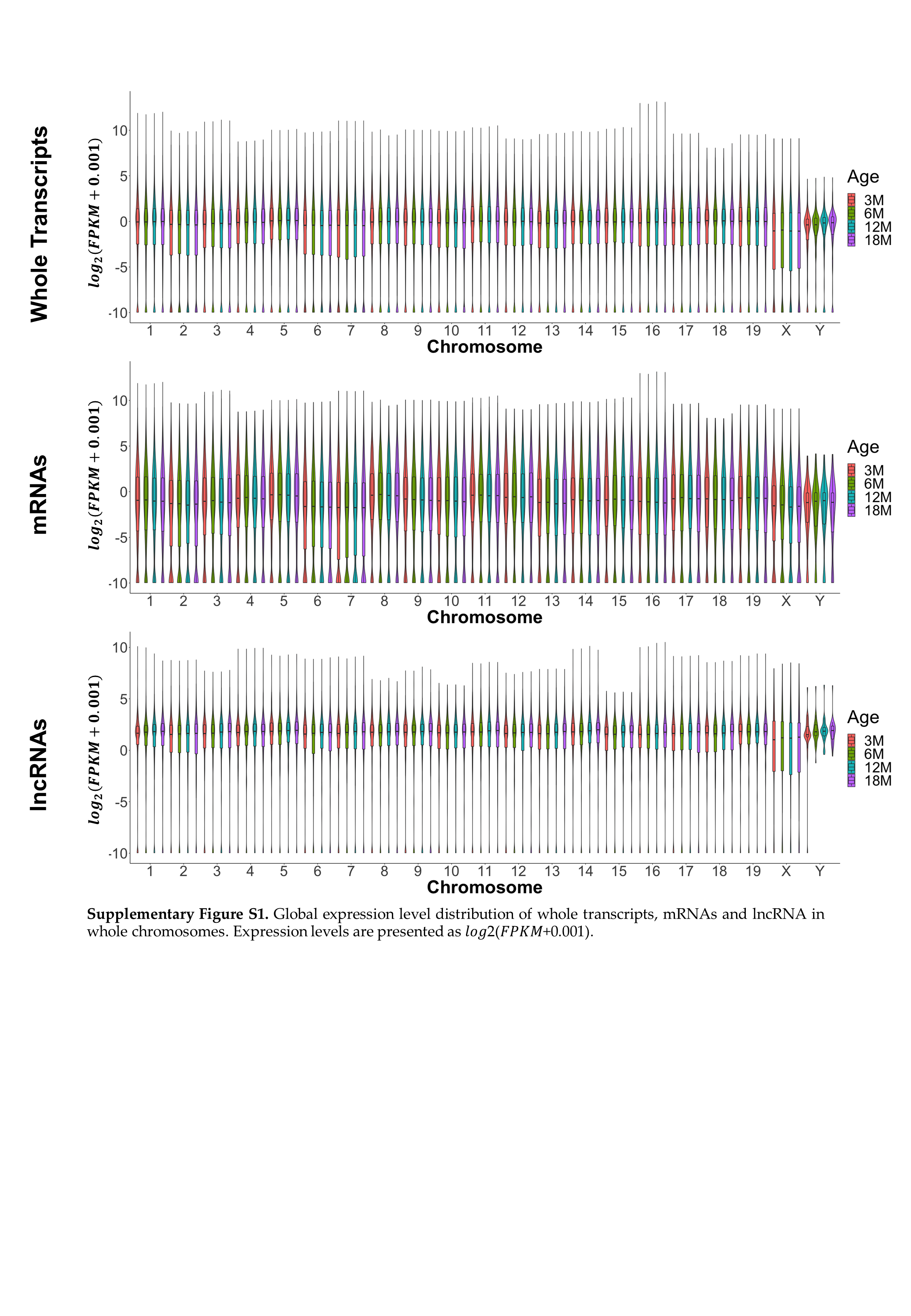

Supplement: Supplementary file 1 [file cells-10-02895-s001.zip › Supplementary Figure S1.TIF]

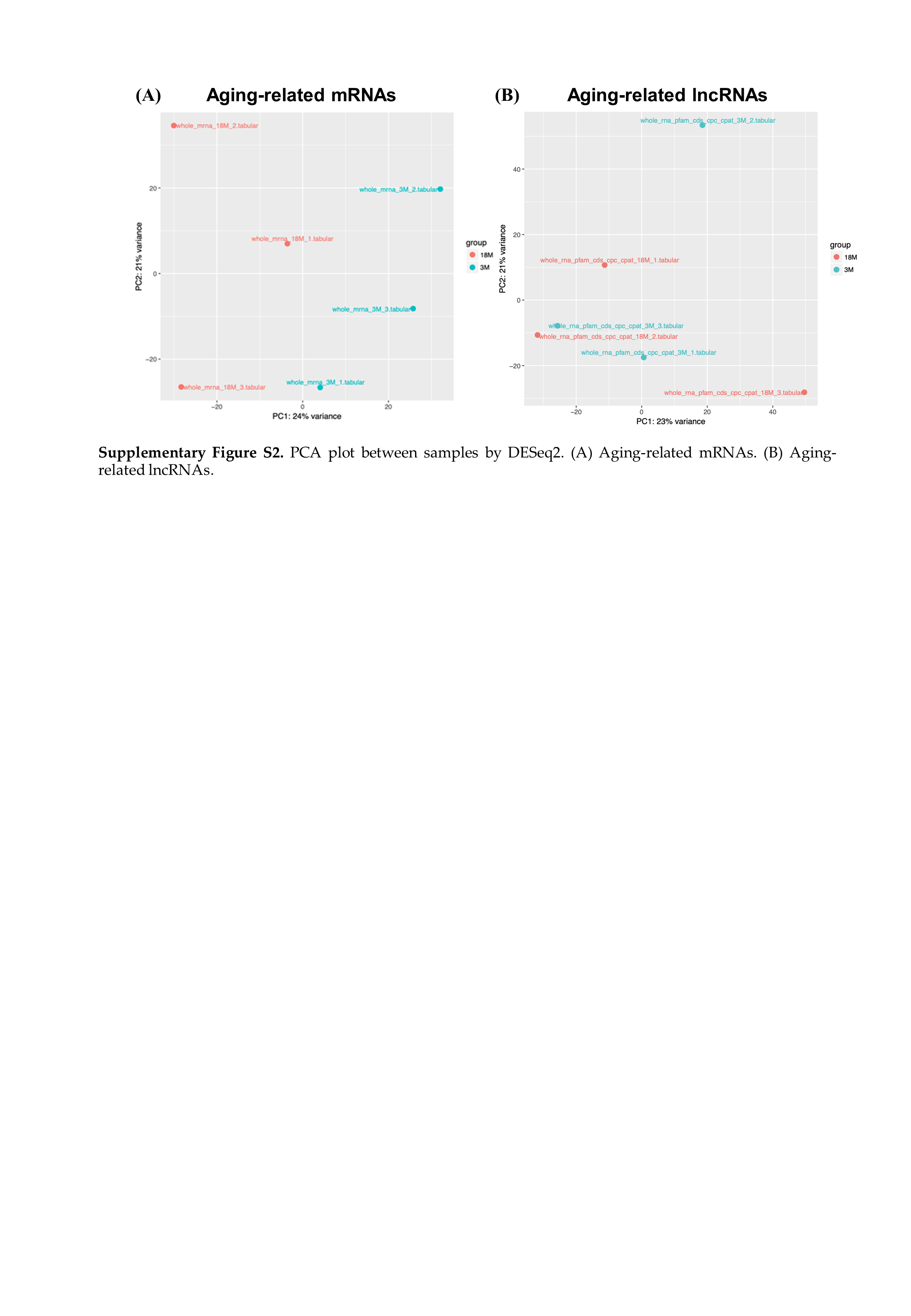

Supplement: Supplementary file 1 [file cells-10-02895-s001.zip › Supplementary Figure S2.TIF]

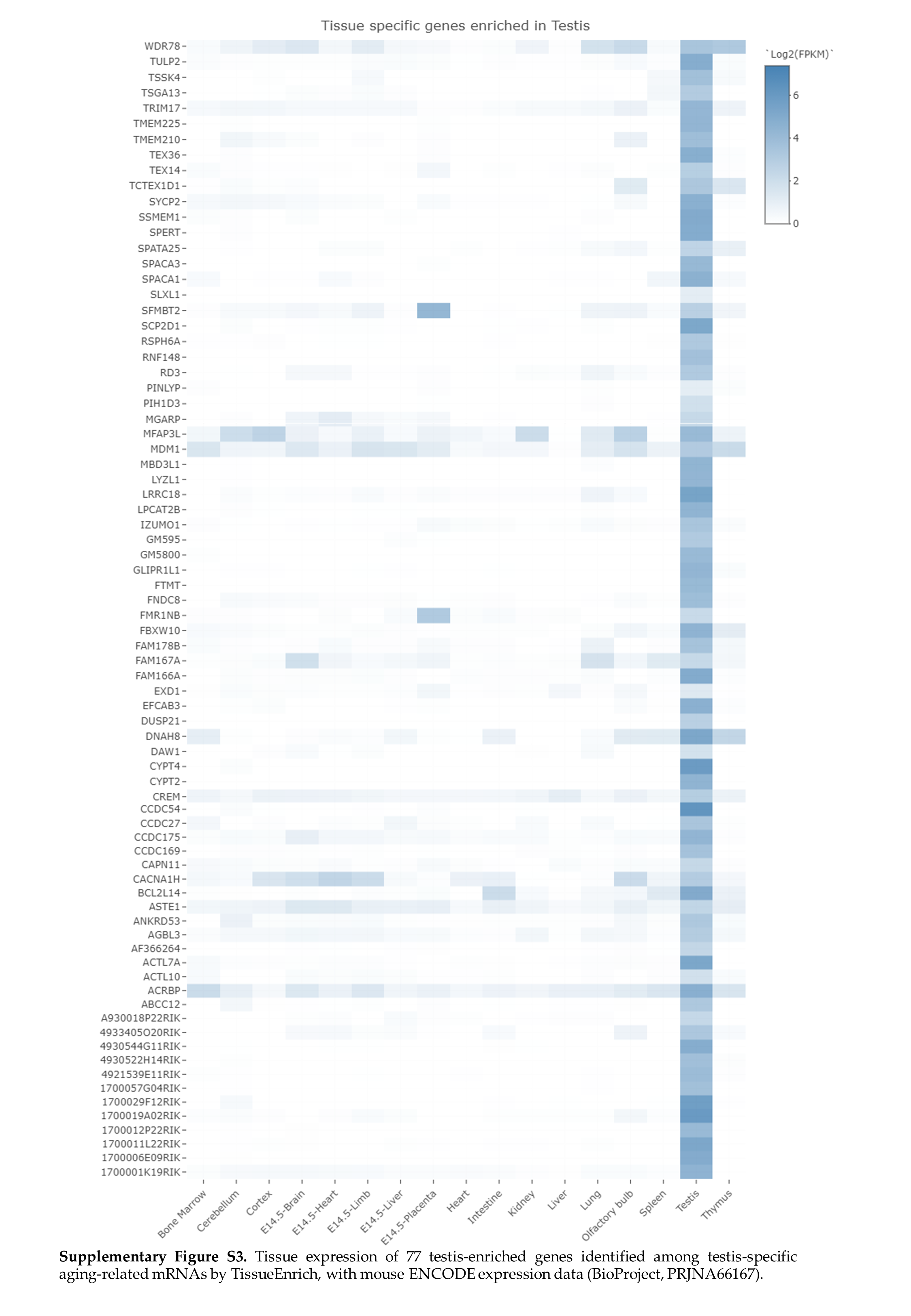

Supplement: Supplementary file 1 [file cells-10-02895-s001.zip › Supplementary Figure S3.TIF]

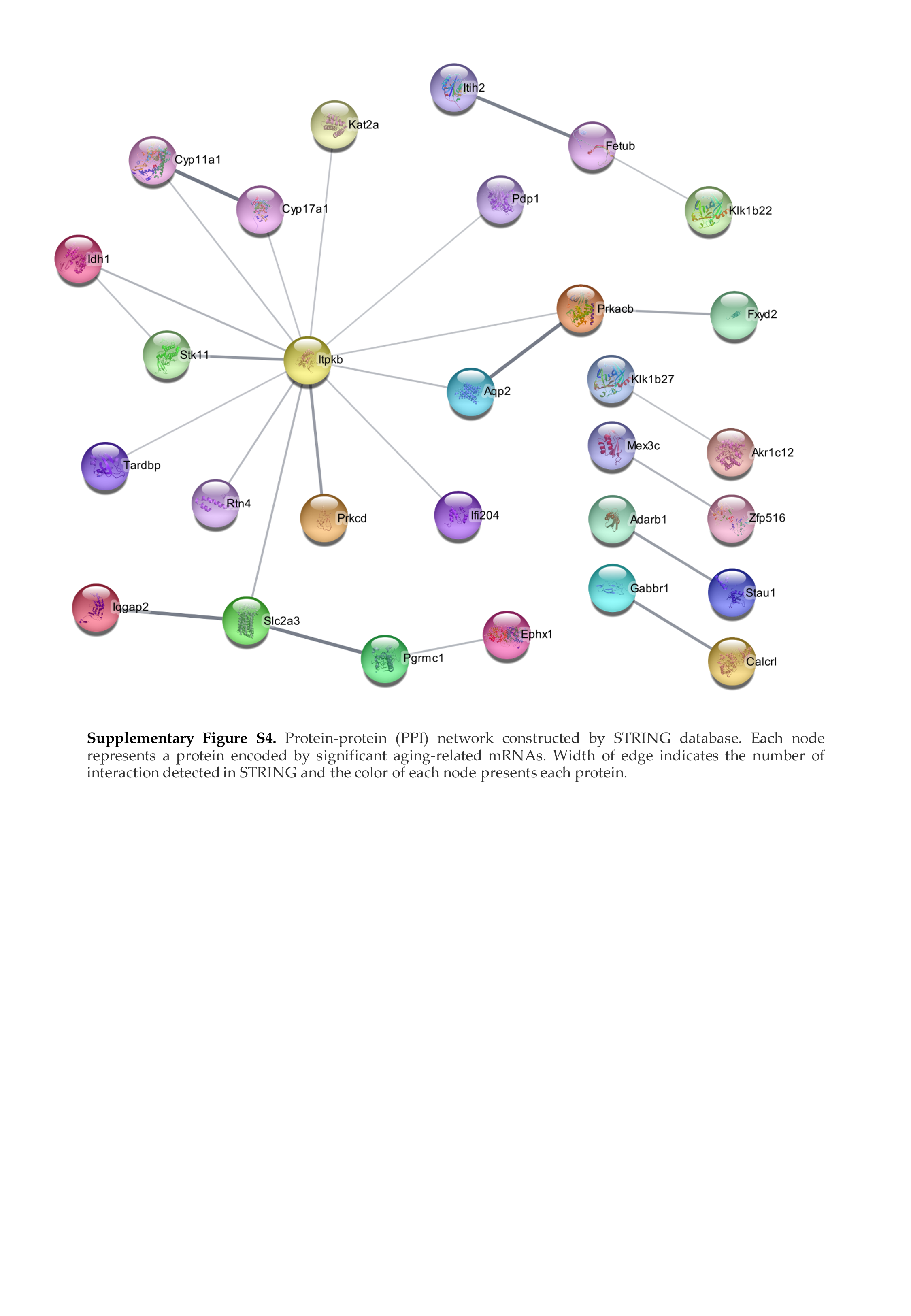

Supplement: Supplementary file 1 [file cells-10-02895-s001.zip › Supplementary Figure S4.TIF]

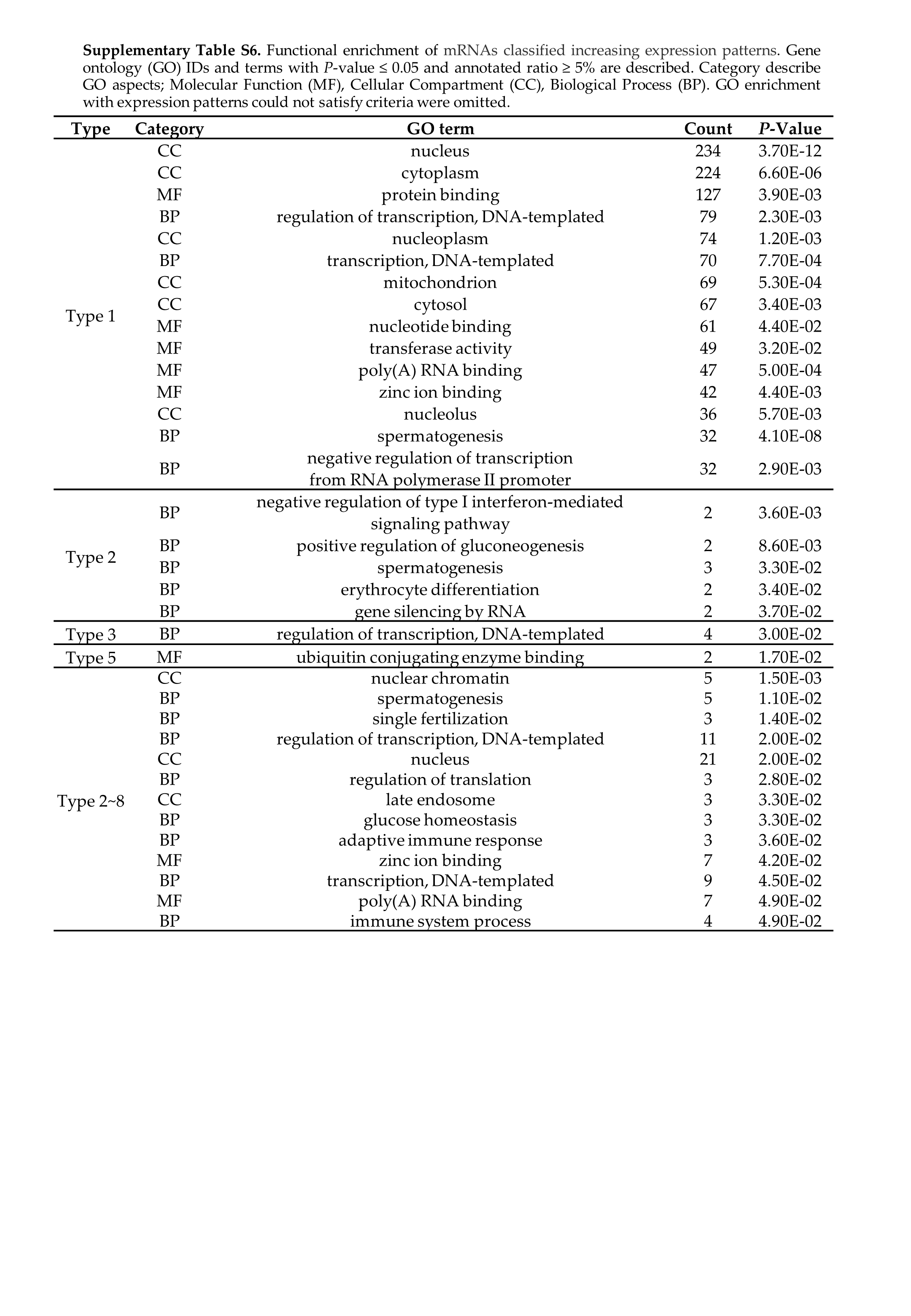

Supplement: Supplementary file 1 [file cells-10-02895-s001.zip › Supplementary Table S6.TIF]

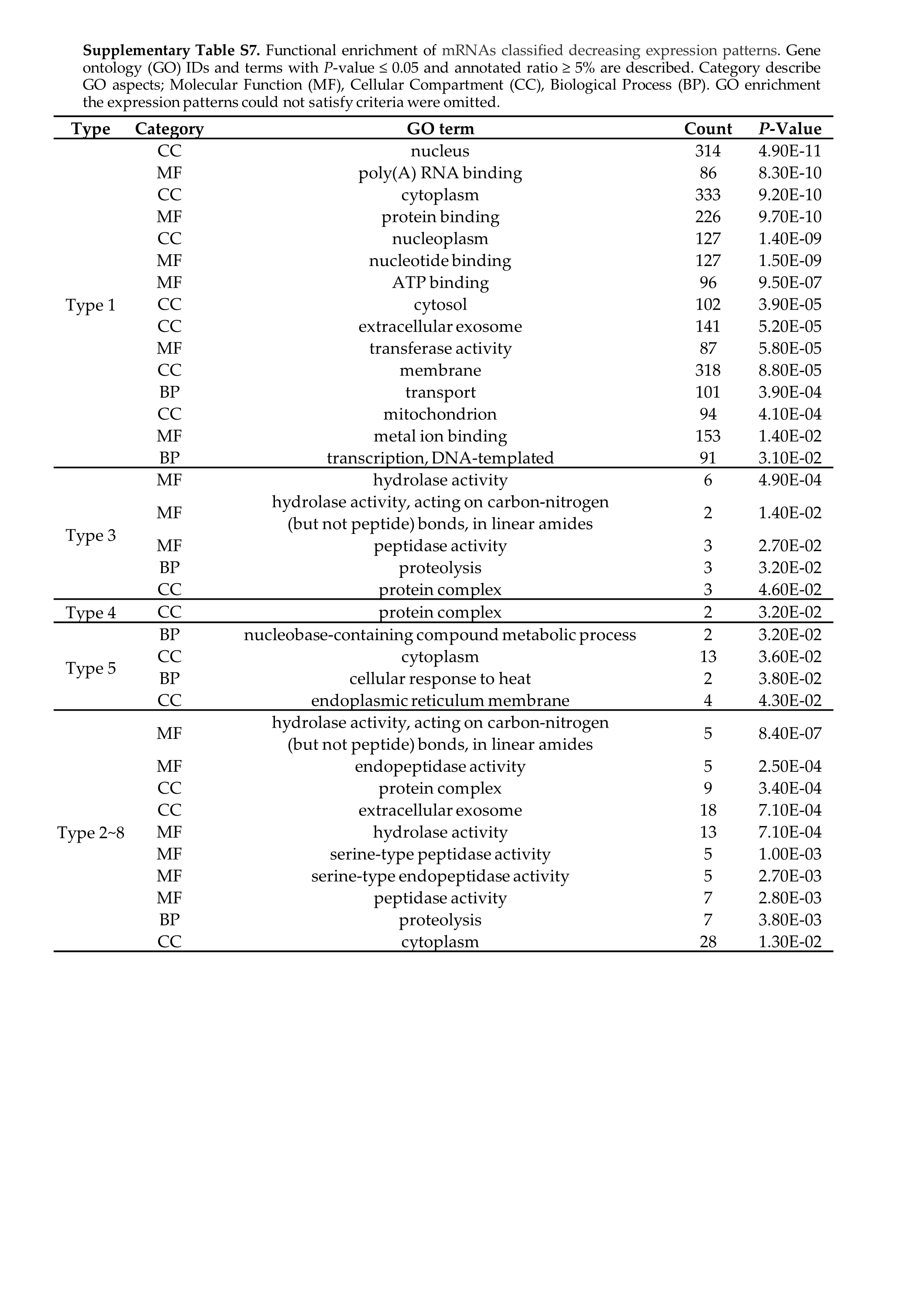

Supplement: Supplementary file 1 [file cells-10-02895-s001.zip › Supplementary Table S7.TIF]

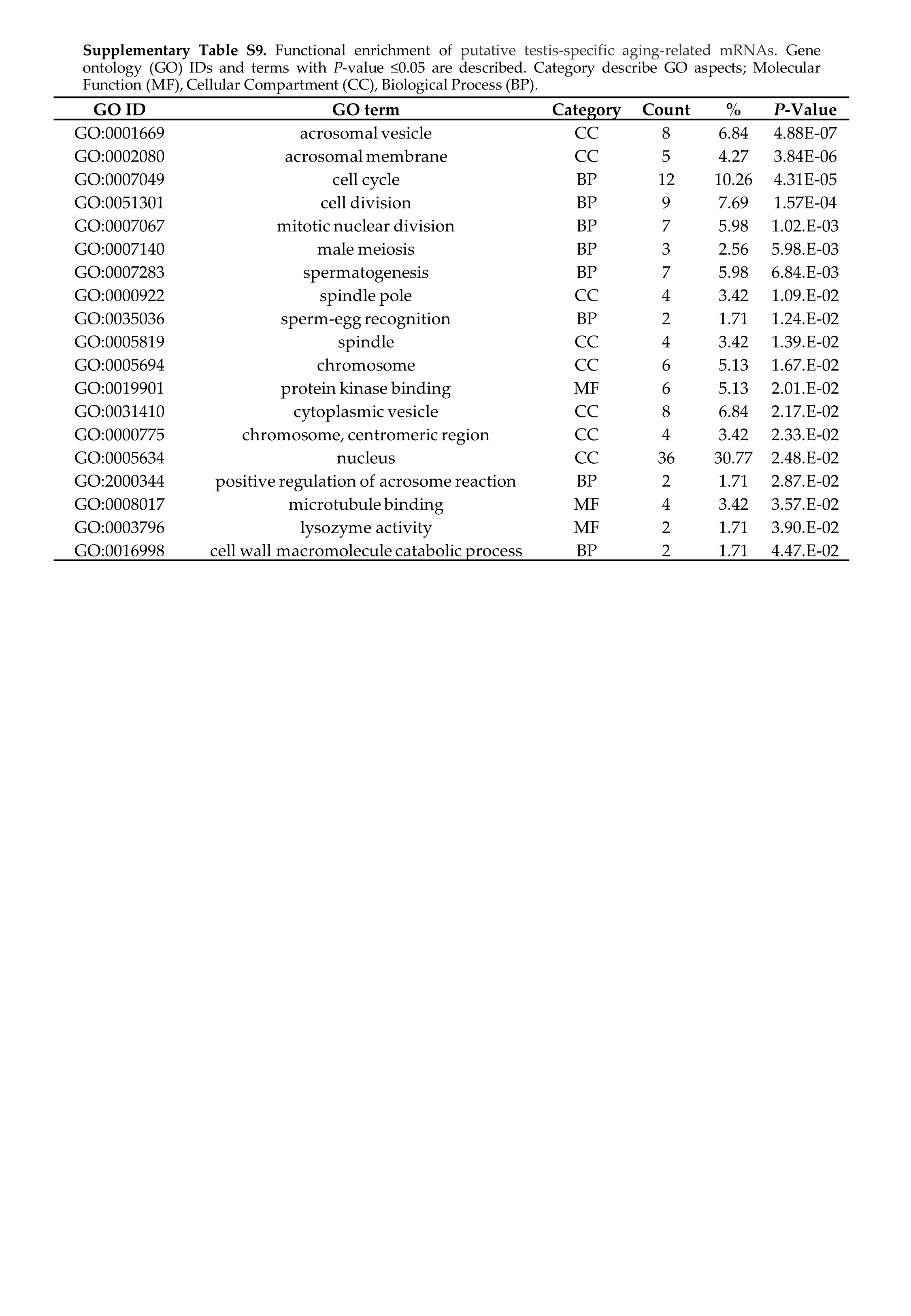

Supplement: Supplementary file 1 [file cells-10-02895-s001.zip › Supplementary Table S9.TIF]
